# Pulmonary Immune Dysregulation and Viral Persistence During HIV Infection

**DOI:** 10.3389/fimmu.2021.808722

**Published:** 2022-01-04

**Authors:** Yulia Alexandrova, Cecilia T. Costiniuk, Mohammad-Ali Jenabian

**Affiliations:** ^1^ Infectious Diseases and Immunity in Global Health Program, Research Institute of McGill University Health Centre, Montreal, QC, Canada; ^2^ Department of Microbiology and Immunology, McGill University, Montreal, QC, Canada; ^3^ Department of Biological Sciences and CERMO-FC Research Centre, Université du Québec à Montréal, Montreal, QC, Canada; ^4^ Division of Infectious Diseases and Chronic Viral Illness Service, McGill University Health Centre, Montreal, QC, Canada

**Keywords:** HIV, HIV reservoir, pulmonary immunity, lungs, alveolar macrophages, CD8 T-cell dysfunction, mucosal immunity, alveolar macrophage (AM)

## Abstract

Despite the success of antiretroviral therapy (ART), people living with HIV continue to suffer from high burdens of respiratory infections, lung cancers and chronic lung disease at a higher rate than the general population. The lung mucosa, a previously neglected HIV reservoir site, is of particular importance in this phenomenon. Because ART does not eliminate the virus, residual levels of HIV that remain in deep tissues lead to chronic immune activation and pulmonary inflammatory pathologies. In turn, continuous pulmonary and systemic inflammation cause immune cell exhaustion and pulmonary immune dysregulation, creating a pro-inflammatory environment ideal for HIV reservoir persistence. Moreover, smoking, gut and lung dysbiosis and co-infections further fuel the vicious cycle of residual viral replication which, in turn, contributes to inflammation and immune cell proliferation, further maintaining the HIV reservoir. Herein, we discuss the recent evidence supporting the notion that the lungs serve as an HIV viral reservoir. We will explore how smoking, changes in the microbiome, and common co-infections seen in PLWH contribute to HIV persistence, pulmonary immune dysregulation, and high rates of infectious and non-infectious lung disease among these individuals.

## Introduction

HIV-1 infection is characterized by chronic immune activation and inflammation, which are predictors of disease progression (1, 2). In fact, HIV+ status has been linked to higher prevalence of age-associated noncommunicable comorbidities, such as cardiovascular and renal disease, where inflammation is believed to be the driving factor (3). Furthermore, chronic inflammatory states persist in PLWH despite virologic suppression with antiretroviral therapy (ART), which has been linked to a higher non-AIDS related morbidity and mortality rate (4, 5). Chronic inflammation and the prevalence of infectious and non-infectious comorbidities is especially pertinent in regards to the lungs. Even on ART, PLWH are 25 times more likely to suffer from pneumonia and are at a higher risk of developing chronic obstructive pulmonary disease or lung cancer than healthy individuals (6–10). Rates of tuberculosis infection and influenza-associated mortality is also higher in PLWH than in general population (11, 12).

Despite the success of ART at inhibiting HIV replication, the major challenge for a functional HIV cure is viral persistence in cellular and tissue reservoirs. An HIV reservoir is an anatomical site harboring cells where a replication-competent proviral DNA with stable kinetic properties persists in its integrated form (13). As a retrovirus, HIV integrates its DNA into the genome of host cells, mainly CD4 T cells (14). After acute infection, the provirus enters a state of post-integration latency whereby viral transcription is reversibly silenced. The lack of viral protein expression on the cell surface protects the cell from detection and elimination by the immune system (15). Cellular HIV reservoirs are established during the early days of infection, whereby HIV seeds different anatomical sites such as lymph nodes, gut-associated lymphoid tissue, the central nervous system, the genitourinary tract and, based on recent evidence, the lungs (16–18).

Early viral presence in the lung, along with high rate of lung diseases in PLWH even after ART initiation raise the question on why and how the lung might serve as another anatomical HIV reservoir and what challenges does it pose on our way to a true functional HIV cure.

## Lung Immunity

Just like the skin and the gastrointestinal tract, the lungs are an interface between the inner body and the outside world. Apart from ensuring adequate gas exchange, the lung must prevent harmful effects of noxious compounds, microbes, and debris on the body. The lungs are designed to facilitate optimal oxygen exchange between the environment and red blood cells. Thus, not only are they highly vascularized, but they also house around 300 million alveoli, covering a surface area of approximately 500 m^2^ – roughly the size of a tennis court (19, 20). This entire area must be kept clean of unwanted airborne particles and is patrolled continuously for foreign invaders – a task executed by the innate and adaptive arms of the immune system.

### Physical and Biochemical Barriers

The human respiratory tract can be divided into the upper and lower parts. The upper portion is composed of the nasal cavity, pharynx, and larynx, while the lower portion includes the trachea and the lung itself, which houses part of the conducting airways (bronchi, bronchioles) that lead into the respiratory zone (the alveoli) (21). The lumen of the airways is lined with epithelium that is made up of ciliated, non-ciliated, and secretory cells (21). These are sealed shut with tight junctions, creating a physical barrier, whose permeability is controlled by transmembrane proteins called claudins (22). This barrier is further reinforced by a layer of airway surface fluid made of mucins - large heavily glycosylated proteins secreted by goblet cells that form a gel-like physical barrier that protects the underlying cells from physical and chemical stressors. Moreover, this barrier acts as an antimicrobial wall, trapping any microbes with which it comes into contact (23). In addition to mucins, airway surface fluid contains a large number of antimicrobial proteins and peptides, such as defensins and lysozymes, which collectively exhibit broad-spectrum antimicrobial activity (24). Airway mucous, along with the trapped microbes and debris within it, is pushed out continuously from the lower lung into the trachea and esophagus by the mucociliary clearance system where ciliated cells move the surface fluid *via* the motive force of their cilia. As we progress from the terminal bronchus into the alveolar sacs, the cellular and airway surface fluid makeup changes. Here, in the alveolar sacs, the epithelial barrier becomes extremely thin and its surface mucus becomes replaced by surfactant, a detergent-like substance that prevents alveolar collapse when we breathe (25). Since gas exchange in this zone is vital, structural damage, air flow obstruction, or uncontrolled inflammation within this tissue can have life threatening consequences (26).

### Sensor Cells of the Innate Immune System

The next tier of the lung’s defense system are sensor cells, which include the aforementioned epithelial cells along with alveolar macrophages (AMs), dendritic cells (DCs), and mast cells (25). In bronchoalveolar lavage (BAL) fluid, AMs make up the majority of this cell pool (~85%) (27). Unlike most immune cells, AMs are largely derived from a unique tissue-resident cell subset that originates from a distinct hematopoietic cell lineage during embryonic development (28, 29). When quiescent, the primary role of AMs is to clear the lung of allergen particles, dead cells, and other debris to maintain tissue homeostasis. They also receive multiple inhibitory signals, such as CD200, transforming growth factor−β (TGF-β) and interleukin−10 (IL−10), from pulmonary epithelium that prevent their unnecessary activation (30). Notably, healthy lung microbiota further supports these anti-inflammatory homeostatic functions (31). However, during an active infection, these processes are interrupted. Once a pathogen is encountered and recognized by one of the aforementioned sensor cells, pro-inflammatory cytokines are released, which immediately initiate an innate immune response. The type of immune response initiated depends on the nature of the pathogen and the type of cytokines it triggers. For instance, in the case of a viral infection, epithelial cells, AMs and DCs begin producing Type I and Type III interferons (IFNs), to limit pathogen spread and induce an antiviral state of the cells in the vicinity (32).

### Engaging the Adaptive Immune System

If the aforementioned mechanisms fail to clear the pathogen, tissue-resident lymphoid cells are recruited. These include innate lymphoid cells, natural killer cells, Natural Killer (NK) T-cells, conventional CD8 and CD4 T-cells, as well as mucosal-associated invariant T-cells (MAIT) and γδ T-cells. Collectively, these cells further enhance direct killing of the pathogen or infected cells and recruit more effector cells from the circulation, such as neutrophils and monocytes, to facilitate infection clearance in its early stages (25). If the pathogen is still not cleared, additional forces of the adaptive immune system are called to action. DCs are the key mediators of this process. Residing under the epithelial layer within the pulmonary interstitium, these cells can extend their dendrites across the epithelial layer to sample antigen from the lumen. Under inflammatory conditions, DCs become activated and begin to transport the microbial antigen to the draining lymph nodes and nearby mucosal associated lymphoid tissues, where they activate naïve and central memory T-cells whose T-cell receptor matches the MHC-peptide complex on DC’s surface (33–35). The efficiency of DCs to activate these cells depends on both the nature of infection and the type of T-cell it encounters. For example, during respiratory influenza virus infection, CD103+ migratory DCs are the most potent activators of naïve virus-specific CD8 T-cells (36, 37).

CD8+ cytotoxic T-cells and CD4+ Th1 cells fight intracellular microbes by killing infected cells, releasing pro-inflammatory and anti-viral cytokines (IFN-γ), and recruiting phagocytes to the infection site (25, 33, 38). CD8+ Tc2 cells and CD4+ Th2 cells fight extracellular parasites *via* granulocyte recruitment, mast cell activation, stimulation of mucus production by goblet cells, and promotion of B-cell class-switching to IgE (25, 38). CD4+ Th17 and CD8+ Tc17 cells are devoted to battling against extracellular bacteria and fungi. These cells amplify neutrophil recruitment, stimulate antimicrobial peptide production by the pulmonary epithelium, and promote B-cell class-switching to opsonizing antibody production (38, 39). B-cells and follicular helper T-cells (Tfh) are crucial against all classes of pathogen. With the aid of Tfh cells, B-cells expand and differentiate. Depending on the activation site and signals they receive from Tfh cells, some will traffic back to the airways and become local IgA producing plasma cells, while others will switch to IgG and home to the bone marrow to provide systemic protection (40). Notably, in a healthy respiratory tract, IgA is the major immunoglobulin and IgA deficient individuals experience higher rates of respiratory infections (20, 41).

Lastly, regulatory T-cells (Tregs) are tasked with resolution of inflammation upon infection clearance. Their job is crucial in collateral damage control caused by pro-inflammatory immune mechanisms. Originating either from the thymus (natural Tregs) or from conventional CD4+ T-cells differentiated in the periphery (induced Tregs), these cells are potent immune suppressors. They downscale immune activation, kill effector T-cells, limit growth factor availability, and promote tissue repair returning the lung back to homeostasis (42–44).

## Pulmonary Immune Dysregulation During HIV Infection

### Acute HIV and Lung Immunity

Within a few hours of the transmission event, HIV begins to replicate in mucosal, submucosal, and draining lymphoid tissues. Notably, lung is an early target of HIV dissemination because it is highly vascularized and houses a very large pool of target cells. Experimental SIV-infections of macaques demonstrate that the virus is seeded into the lungs shortly after intravenous infection (45–47). In fact, SIV replication in BAL cells of pigtailed macaques can be detected as early as 7 days post-inoculation, peaking at 10 days during acute infection (46). Interestingly, the CCR5-tropic HIV strain, known as HIV-1Bal, was originally isolated from the lungs (48). Viral quasispecies specific to this tissue and distinct from the circulation have also been reported (49, 50).

Upon reaching the lung, HIV is seeded into multiple cell types: CD4 T-cells, DN T-cells, and AMs. Although HIV preferentially infects CD4 T-cells, which account for ~6% of total BAL cells in healthy non-smokers, AMs, which account for ~85% of total BAL cells, are also infected (27, 51–53). Although data on pulmonary immune perturbations during primary acute HIV infection is scarce, *in vitro* experiments on human lung lymphocytes and *in vivo* animal SIV models suggest that during the acute phase of infection pulmonary interstitial CD4 T-cells are more severely and rapidly depleted compared to the blood compartment that is largely due to CCR5+ memory CD4 T-cells’ high susceptibility to CCR5-topic HIV-1 infection, which make up the vast majority of the lung CD4 T-cell pool (54, 55).

Most acute HIV-1 infections are caused by the CCR5-tropic strain (T-tropic strain), which preferentially targets T-cells. Although it’s not very efficient at infecting macrophages on its own, its infectivity is enhanced significantly during cell-to-cell contact between AMs and infected CD4 T-cells, as has been recently shown by Schiff and his group, suggesting that AM HIV entry during acute infection is CD4 T-cell dependent (53). Within the AM cell pool (CD206+) two subsets have been identified based on size (FSC) and granularity (SSC) – small and large AMs (56). Human *ex vivo* AM analysis shows that HIV preferentially infects monocyte-like small AMs, which show higher pro-inflammatory gene expression and greater phagocytic capacity compared to large AMs (56, 57). In addition to infecting AMs, we have also shown in a humanized mouse model of early HIV infection that the virus is preferentially seeded within lung DN T-cells, a rather novel lung HIV reservoir cell subset, which is enriched in the lungs of both HIV+ and seronegative individuals compared to other tissues (58).

In about two thirds of cases with primary HIV infection, individuals experience flu-like symptoms such as fever, chills, and swollen lymph nodes. Some individuals also suffer from respiratory symptoms such as a sore throat or a dry cough, suggesting engagement of the pulmonary innate immune response (59). Massive CD4 T-cell apoptosis during the acute infection phase is accompanied by profound immune activation, caused by release of apoptotic microparticles into the bloodstream (1, 60). Furthermore, in addition to the attack on human immune cells, HIV also compromises the integrity of the lung epithelial barrier by infecting human bronchial epithelial cells, decreasing their expression of E-cadherin, and increasing paracellular permeability (61, 62).

### Untreated Chronic HIV and the Lung Immunity

At this point, viral reservoirs in deep tissues are already well established, including the reservoir in the lung. In fact, SIV-models show that during the asymptomatic phase of infection, there is no correlation between plasma and BAL fluid viral loads (46). This is further supported by human phylogenetic studies, which demonstrate HIV lung reservoir compartmentalization in untreated patients (63, 64). Furthermore, whole lung tissue biopsies of untreated HIV-infected persons harbor distinct viral quasispecies compared to those found in their blood and lymphoid tissues suggesting that the HIV reservoir may be replicating and evolving locally in that anatomical site, rather than solely spreading from the circulation (49, 50). 

Prior to introduction of ART, lung disease was the leading cause of death in people living with HIV (PLWH) (65). As seen in the very first AIDS reports of *Pneumocystis jirovecii pneumoniae* infections in gay men in the 1980s, previously rare fungal pulmonary infections became a staple AIDS-defining illness observed in severely immunosuppressed patients (66, 67). Untreated PLWH are also at a higher risk of developing recurring bacterial pneumonia, whose rate is inversely proportional to the patients’ CD4 T-cell counts (68). They are also more prone to cancer development, such as lung cancer, compared to persons without HIV infection (69, 70).

The dysregulated pulmonary immune environment in PLWH may facilitate the development of such lung pathologies. Destruction of lung parenchyma, pulmonary inflammation, and emphysema are recognized complications of HIV infection. Up to 60% of untreated PLWH present with lymphocytic alveolitis, characterized by infiltration of B-cells, gamma-delta T-cells, as well as CD4+ and CD8+ T-cells into the lung, which is observed in absence of any respiratory symptoms (71–74). Both their pulmonary CD8 and CD4 T-cells show 2- to 3-fold greater expression of HLA-DR and CD38, which are markers of immune activation, compared to seronegative adults (75). Neff and colleagues have also shown that in untreated PLWH with lymphocytic alveolitis, lung HIV-specific CD4+ and CD8+ T-cells show impaired proliferative capacity, which is caused by high expression levels of PD-1, a classic exhaustion marker that dampens T-cell receptor signaling (71, 76). Furthermore, the lung cytokine milieu of PLWH is disrupted. Jambo et al. have shown that BAL fluid taken from ART-naïve HIV+ study participants have increased concentrations of RANTES and TNF-β and a shift towards MIP-1β, MCP-1, and IP-10 signaling network (77). Notably, RANTES is a lymphocyte chemoattractant, shown to a play a role in lung CD8 T-cell recruitment in other viral lung infections. These CD8 T-cells can in turn produce TNF-β, a potent pro-inflammatory cytokine that promotes vascular cell adhesion, chemokine production, and further immune cell infiltration into the tissue (78–80). IL-6 is another pro-inflammatory player, whose levels are associated with higher HIV RNA levels and has repeatedly been shown to be produced by monocytes and macrophages in response to HIV (81, 82). *In vivo* non-human-primate models further show that IL-6 expression in pulmonary interstitial macrophages of SIV-infected animals is positively correlated with monocyte turnover and lung tissue damage (83). As mentioned previously, HIV has also been shown to impair pulmonary epithelial integrity by decreasing expression of cell-to-cell adhesion molecules and promoting further release of pro-inflammatory mediators by these cells, thus accelerating the decline in lung function (61).

Chronic pulmonary inflammation further leads to increased production of matrix metalloproteinases (MMPs), a family of endopeptidases that can degrade elastin and collagen fibers (84). Notably, both elastin and collagen degradation products act as immune cell chemoattractants: while elastin fragments recruit monocytes, collagen fragments attract neutrophils (85). Collectively, MMPs play a role in tissue repair and modulate the immune response. *In vitro* experiments on primary human airway basal cells have shown that HIV infection can force these cells to acquire a destructive phenotype *via* upregulation of MMP-9 through activation of MAPK signaling, thus potentially contributing to emphysema development in PLWH (86).

On the other end of the scale between inflammation and wound repair, HIV leads to higher levels of TGF-β – an anti-inflammatory cytokine produced by Tregs and alveolar macrophages. Because PLWH experience persistent low-grade chronic inflammation, that is in part caused by bacterial translocation across the gastrointestinal mucosal barrier, the immune system tries to counteract it *via* anti-inflammatory cytokines, such as TGF-β (87, 88). Notably, TGF-β levels are significantly higher in PLWH compared to seronegative individuals and remain elevated regardless of ART treatment and viral load suppression (89). TGF-β can downregulate inflammatory processes by promoting Treg expansion and inhibiting effector T-cell function, as well as drive collagen deposition by fibroblasts as part of the normal wound repair process (90, 91). In PLWH long-term TGF-β elevation may contribute to irreversible tissue fibrosis of the gut, secondary lymphoid organs, and the lung (92–95). One study has also shown higher TGF-β production by AMs from PLWH compared to AMs from healthy donors, which the authors believe to be implicated in impaired IgG secretion in the alveoli (96). AMs from PLWH also show a pro-inflammatory phenotype, higher TNFα production, and impaired phagocytic ability, which in turn leads to poor pathogen clearance (57, 97–99). Untreated HIV infection also leads to loss of anti-inflammatory CD163+CD206+ AMs (100). Furthermore, AMs from SIV infected macaques show elevated levels of PD-1 which positively correlates with plasma viral load, suggesting that higher PD-1 expression on AMs may be associated with disease progression (101).

### ART-Treated HIV and Lung Immunity

Although introduction of ART has greatly reduced the rate of opportunistic infections and improved the quality of life of PLWH, it does not fully restore all of immune perturbations caused by HIV infection. Instead, the spectrum of most prevalent pulmonary diseases had shifted from opportunistic infections to chronic illnesses, such as emphysema, chronic obstructive pulmonary disease (COPD), pulmonary fibrosis, and lung cancer which are discussed in more detail in a later section. Notably, although ART had greatly reduced the rate of lung infections in PLWH, they still occur more frequently in HIV infected individuals compared to the general population (7, 102).

Viral control after ART-initiation has a significant impact on lung immunity. Lymphocytic alveolitis resolves, CD8 T-cell numbers decrease, CD4 T-cell count improves, and thus CD4:CD8 T-cell ratio is ameliorated (103, 104). Notably, lung CD4 T-cell repopulation likely occurs due to local expansion of the tissue resident subset, as seen by higher Ki67 expression in BAL CD4 T-cells 1 month after starting therapy (104). Twigg et al. have also demonstrated that level of CD38 and HLA-DR on BAL lymphocytes decrease significantly after the first 6 months of therapy, especially on CD8+ T-cells (105). However, as our team has recently demonstrated, levels of HLA-DR+CD38+ BAL CD4 T-cells remain higher in ART-treated PLWH compared to healthy controls (17).

Twigg and his group also show that intracellular levels of INF-γ, TNF-β, and IL-2 in alveolar CD4+ and CD8+ T-cells, measured after mitogenic stimulation with the superantigen staphylococcal enterotoxin B, decline significantly in ART-treated PLWH (105). Similar reports were made on extracellular levels of inflammatory cytokines in BAL fluid, such as INF-γ, IL-6, and INF-γ inducible chemokines like IP-10 (105–107). In contrast, Knox et al. showed that although BAL CD4 T-cell infection rate decreases with introduction of ART, both CD4 and CD8 T-cell polyfunctional profiles (INF-γ, TNF-α, IL-2) after mitogenic stimulation remain relatively unchanged (104). Importantly, this was not the case in the peripheral blood, where CD8 T-cells showed a marked improvement in polyfunctional cytokine secretion in response to a stimulus (104). These findings further highlight that immune restoration in lung tissue during ART is incomplete, which can help explain ongoing susceptibility of treated PLWH to pulmonary infections (108, 109).

Apart from pro-inflammatory mechanisms, anti-inflammatory immune functions also remain dysregulate during ART. PLWH show higher levels of CD39+CD73+Tregs in their BAL fluid compared to peripheral blood, while no such difference is observed in seronegative controls (17). Importantly, these immunosuppressive cells can act as a double-edged sword. They can help resolve inflammation during acute lung injury, but they are also capable of promoting tumor cell survival, angiogenesis, and fibrosis (110, 111).

**Figure 1:**
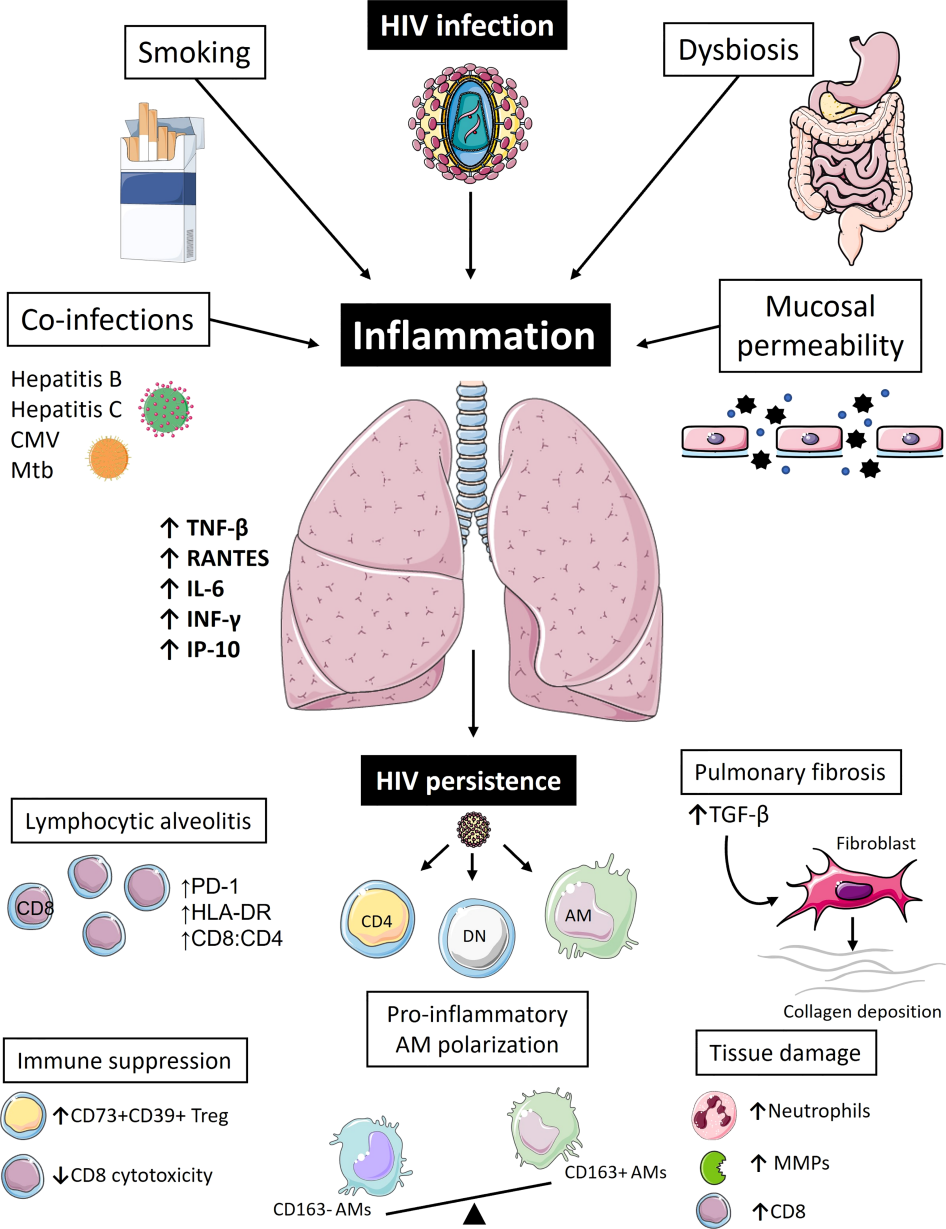
Pulmonary inflammation drives immune dysregulation and reservoir persistence in people living with HIV. Even during the era of antiretroviral therapy (ART), people living with HIV (PLWH) continue to suffer from high burdens of pulmonary illnesses. Inflammation is likely the biggest driver of pulmonary pathologies and lung HIV reservoir persistence in these individuals. Apart from the virus itself, which is seeded into the lung within the first few weeks of infection, other factors also contribute to pulmonary immune perturbations in PLWH even during ART, such as smoking, co-infections, changes in the microbiome, and compromised integrity of mucosal barriers. Collectively, these fuel chronic inflammatory state and pulmonary immune activation characterized by high levels of pro-inflammatory cytokines (RANTES, TNF-β, IFN-γ, IL-6, IP-10), which in turn lead to immune cell recruitment to the lung tissue, typically presenting as CD8 T-cell lymphocytic alveolitis. These CD8 T-cells appear as functionally impaired and fail to remove lung HIV reservoir that continues to persist in mucosal CD4+ T-cells and CD4-CD8- Double Negative (DN) T-cells, as well as alveolar macrophages (AMs). A vicious cycle of immune activation and residual viral replication ensues further exacerbating pulmonary immune abnormalities, such as pro-inflammatory AM polarization, extracellular matrix destruction caused by increased production of matrix metalloproteinases (MMPs), further CD8 T-cell recruitment, and increased neutrophil count. In an attempt to counteract this inflammatory process, immunoregulatory arm of the immune system could further contribute to increased risk of pulmonary co-morbidities: accumulation of immunosuppressive regulatory T-cells (CD73+CD39+Treg) might be implicated in higher lung cancer risk and higher levels of TGF-β could be the primary driver of irreversible pulmonary fibrosis. [Images adapted from Servier Medical Art licensed under CC 3.0 (smart.servier.com)].

AM dysfunction is yet another factor contributing compromised lung immunity in PLWH on ART. Collini et al. have shown that AMs from ART-treated PLWH have defects in microbicidal mechanisms, mediate by HIV’s gp120 protein, which could inhibit macrophage apoptosis induction, caspase activation, and mROS-dependent pneumococcal killing (112). These cells also remain subjected to chronic oxidative stress despite ART. Yeligar et al. have shown that BAL fluid from treated PLWH has higher H_2_O_2_ concentration (113). Furthermore, their AMs have lower expression levels of proliferator-activated receptor (PPAR)-γ, an important player in combatting oxidative stress during acute lung injury, higher expression levels of expression of NADPH oxidases, which further promote oxidative stress and inhibit phagocytosis, and higher levels of TGF-β, a big driver of tissue fibrosis (113–116). Impaired AM phagocytic capacity and skewing in polarization has been further emphasized by Akata et al. (117). Using BAL samples from PLWH and healthy controls, they have demonstrated that out of the four macrophage subsets (non-polarized: CD40-, CD163-; M1: CD40+, CD163-; M2: CD40-, CD163+; double-polarized: CD40+, CD163+) the double-polarized subset has the highest phagocytic capacity. Notably, this subset was significantly diminished in HIV+ COPD- individuals, while the non-polarized subset, which had the lowest phagocytic capacity, was enriched (117). The collective effects of HIV on pulmonary inflammation are summarized in **Figure 1**.

### ART Toxicity

Interestingly, there are several studies which suggest that ART toxicity might also play a role in immune dysregulation in PLWH. *In vitro* studies have shown that nucleoside reverse transcriptase inhibitors can decrease mitochondrial DNA content and complement-mediated phagocytosis in human monocyte-derived macrophages (118). In addition, Korencak et al. looked at CD4 T-cells from treated and untreated PLWH, as well as healthy subjects, showed that ART improves these cells’ metabolic phenotype but not the respiratory impairment, especially in patients receiving integrase inhibitors. Furthermore, they show that CD4 T-cells treated with dolutegravir and elvitegravir shift the cytokine response of these cells from a polyfunctional one to a TNF-α dominated one (119). A similar observation was made by Bowman and colleagues, which demonstrate that human macrophages exposed to tenofovir disoproxil fumarate and emtricitabine have lower mitochondrial mass and increased lipid uptake (120). These studies can help explain the observations made by Correa-Macedo et al. showing the potential adverse effect of ART on transcriptional response of AMs to *Mycobacterium tuberculosis* (*Mtb*). In their recent study, they have isolated AMs from healthy controls, PLWH on ART, as well as seronegative participants taking pre-exposure prophylaxis (PrEP). Notably, AMs from HIV+ and HIV- PrEP study groups both had a weaker transcriptional response when challenged with *Mtb* compared to AMs from healthy controls. Furthermore, AMs from HIV- PrEP and HIV+ donors showed no change in chromatin state upon challenge, unlike AMs from healthy controls which displayed a significant change in chromatin accessibility (121). Collectively, these findings suggest that although ART greatly improves the quality of life of PLWH and does restore many of their immune parameters it does not come without side effects. Some regimens may decrease mitochondrial function and elicit a pro-inflammatory immune cell profile, thus partially contributing to chronic inflammation in treated individuals.

## Accelerated Pulmonary Co-Morbidities During HIV Infection

### Accelerated Immune Aging

Many age-related co-morbidities occur in PLWH at a younger age than in the general population. This premature onset of age-related illnesses has been largely attributed to chronic inflammation and immune activation, which in turn leads to accelerated immune aging, now known as ‘inflammageing’ (4, 122). Inflammageing is characterized by high level of circulating pro-inflammatory cytokines (IL-6, IL-8, TNF-α, and IFN-γ), telomere shortening, cell senescence (loss of CD28 and increased expression of CD57 and KLRG1 on T-cells), mitochondria dysfunction, and changes in microbial composition of the microbiota, all of which have been documented in PLWH (122–125). Consequently, this accelerated immune aging affects the lungs. Leung et al. had conducted several studies on telomere shortening in PLWH (126, 127). They have shown that telomeres in circulating leukocytes of PLWH are significantly shorter than in seronegative study participants. Furthermore, their telomere length correlated with severity of poor lung function determined by forced expiratory volume (126). Notably, in their more recent publication, which included HIV+ participants with good immune recovery and undetectable viral loads, the telomere length of their small airway epithelial cells was significantly shorter than in healthy controls, even after accounting for cigarette smoke exposure (127). Moreover, HIV+ participants in that study were on average 4 years younger than the seronegative ones.

### HIV and COPD

COPD is a progressive inflammatory lung condition characterized by airway obstruction, inflamed mucous membranes and alveolar damage and is the third leading causes of death worldwide with 2 million people affected in Canada alone (128, 129). Both smoking and HIV are independent risk factors for COPD development (130–132). Furthermore, COPD has often been proposed to be a disease caused by accelerated immune aging (133, 134). A recently published study conducted by Córdoba-Lanús et al. monitored telomere length of patients with COPD over a 10 year period and found an association between accelerated telomere shortening and progressive decline in lung function, such as worsening of gas exchange and lung hyperinflation in COPD patients (135).

HIV-associated COPD also further dysregulates lung immune cell function. Popescu et al. have documented that HIV+COPD+ individuals show severe CD4 T-cell loss in their BAL fluid, mediated by Fas-dependent activation-induced cell death. Their BAL CD4 T-cells also show poor HIV-specific immune response and loss of polyfunctionality compared to HIV+COPD− participants (136). Curiously, unlike lung mucosal CD4 T-cells, BAL CD8 T-cells in these individuals maintained their HIV-specific function. Moreover, increased CD8 T-cell cytotoxicity has been documented previously in COPD patients, which might contribute to this condition’s highly tissue destructive phenotype (137).

### HIV and Lung Cancer

Lung cancer is yet another leading cause of death in PLWH (138). Notably, HIV and COPD are both risk factors for lung cancer development even after accounting for smoking status (139–141). Increased lung cancer risk in PLWH has been attributed to acute inflammatory insults caused by lung infections, chronic low-grade inflammation, CD8 T-cell dysregulation, compromised integrity of pulmonary epithelium, and changes in lung microbiome (142–145). Accelerated immune aging may also play a role. Klugman and colleagues have shown that in the United States PLWH were diagnosed with non-small cell lung cancer at a younger age compared to seronegative participants, with lower median survival time especially among those with a low CD4/CD8 ratio and high viral loads (146). Exhausted CD8 T-cells likely contribute to worse lung cancer outcomes in PLWH, especially in those with multiple co-infections which exacerbate this exhausted phenotype further (147, 148). Given the importance of these cells in fighting cancer, their exhaustion leads to decreased responsiveness to stimuli, low cytotoxicity, poor IFN-γ secretion, and thus compromised ability to kill tumour cells (149–151).

### HIV and Pulmonary Fibrosis

The high rates of pulmonary fibrosis in PLWH compared to those in the general population could be attributed to increased TGF-β levels (95, 152). TGF-β levels are significantly higher in PLWH compared to seronegative individuals and remain elevated regardless of ART treatment and viral load suppression (89). Because TGF-β can drive collagen deposition by fibroblasts it has been implicated in irreversible tissue fibrosis of the gut, secondary lymphoid organs, and the lung in PLWH (92–95). A 2017 multi-center Lung-HIV study has shown that fibrotic lung changes have been observed in 29% of HIV-infected participants, which correlated with viral load but not ART treatment status or CD4 T-cell count (95). Of note, high levels of TGF-β in PLWH might contribute to poor non-small lung cancer outcomes, as it has already been documented in seronegative individuals, although a direct link between TGF-β levels in PLWH and lung cancer outcomes is yet to be established (96, 153–155).

### HIV and Pulmonary Emphysema

In contrast to pulmonary fibrosis, emphysema is characterized by higher lung compliance, increased lung volume and lower expiratory flow rate (156). Increased risk of PLWH of pulmonary emphysema was reported as early as the 1980s (157). More recent studies further confirmed that HIV status is a risk factor for emphysema development independently of smoking (158, 159). Some of the mechanisms in PLWH that likely contribute to this risk have been mentioned previously. These include CD8 T-cell accumulation, increased oxidative stress, AM activation, and increased production of MMPs caused by chronic lung inflammatory state, which can subsequently lead to extracellular matrix destruction (85, 86, 159). Attia et al. have further demonstrated that participants in their HIV+ study group were more likely to have a greater portion of their lung to be affected and were more likely to be diagnosed with COPD compared to seronegative controls diagnosed with emphysema. Additionally, in their HIV+ study arm, low CD4 T-cell counts and high soluble CD14 levels were linked with disease severity, supporting the notion that immune activation in PLWH contributes to the risk of pulmonary emphysema development (158). Whether ART initiation decreases the risk of emphysema development remains rather unclear. Emphysema rates in the pre-ART era were reported to be 15% in PLWH *versus* 2% in the general population (160). In ART-treated patients, the reported incidence rates are even higher. Guaraldi et al. have reported that, of 1,446 HIV-infected patients on ART in their cohort, nearly 50% had evidence for emphysema and/or bronchiolitis based on thoracic computed tomography (CT) scans, with 13% showing signs of bronchiolitis, 19% showing emphysema, and 16% having both (161). Furthermore, among ART-treated HIV-infected participants recruited by Leung et al., emphysema progression was not associated with peripheral CD4 cell counts or CD4:CD8 ratio, HIV viral load, ART classes or duration of ART exposure (162). As proposed by others, the increase in incidence rate of chronic inflammatory conditions in the ART era, of not just emphysema and COPD but also diabetes and cardiovascular disease, can largely be explained by improved life expectancies in treated PLWH, giving them more time to develop these co-morbidities (163, 164).

### Role of Smoking in Pulmonary Co-Morbidities

In a nationwide population-based cohort study conducted, Helleberg and others reported that both all-cause and non-AIDS-related mortalities are higher among HIV+ smokers compared with HIV+ non-smokers. They also highlight that smoking PLWH lose more life-years to smoking than to HIV itself (12.3 years versus 5.1 years respectively) (165). Furthermore, they show that smoking-associated risk of death was 61% among PLWH compared to 34% among healthy controls. Notably, risk of death among former smokers was reduced by 40% compared to current smokers. Around 70% of myocardial infarctions and 27% of cancers in PLWH are related to their smoking status (166). Importantly, unlike with myocardial infarction risk, cancer risk could remain elevated in former smokers even 5 years after smoking cessation (166).

Smoking further increases the risk of COPD and emphysema in PLWH (159, 167). Notably, pulmonary emphysema is more prevalent in HIV+ smokers compared to seronegative smokers and is often developed at a younger age, which could be partially attributed to immune dysregulation of AMs in PLWH (159). Indeed, previous *ex vivo* human studies show an increase in MMP expression in both AMs and epithelial lining fluid in HIV positive smokers with early emphysema compared to HIV negative smokers with the same lung condition (168). Previous reports also show that smoking can activate cytotoxic CD8 T-cells, which can in turn exacerbate pulmonary injury (169–171).

## Lungs as an HIV Reservoir

### Lungs Provide Ideal Grounds for HIV Spread

As organs, the lungs possess several features that may contribute to HIV reservoir establishment, several of which stem from their anatomy. Similarly to the gastrointestinal tract, a confirmed and well-studied HIV reservoir site (172), the lungs are an extension of the external environment. They are constantly exposed to external particles and airborne microorganisms. The antigen load in the lungs is therefore quite high. Furthermore, although lymphocytes make up only 10% of the BAL cell pool, the lymphocyte count in pulmonary interstitium is comparable to that of peripheral blood, with as many as 10 X 10^9^ cells (7). High antigen load can in turn promote activation of these lymphocytes and other immune cells, consequently supporting HIV replication and continuous reservoir replenishment in the lung.

Because the lungs carry out the vital function of gas exchange, they are highly vascularized. Their high blood flow, cell proximity, surface area, and small arteriole size could further aid in HIV cell to cell spread and reservoir compartmentalization (7). Furthermore, the lungs of HIV-infected persons harbor distinct viral quasispecies that are tissue specific suggesting that HIV reservoir may be replicating locally in that anatomical site, rather than spreading from other infected tissues (49, 50).

### HIV Persists in Multiple Lung Immune Cell Types

Early viral presence in the lung, along with high rate of co-morbidities caused by lung diseases in PLWH even after ART initiation, support the notion that lungs serve as another anatomical HIV reservoir. Our team previously assessed HIV persistence in the pulmonary milieu in individuals under long-term treatment (median 9 years) (17). We found that total HIV DNA in BAL CD4 T-cells was significantly higher than in peripheral blood mononuclear cells. Moreover, the lungs were enriched in activated memory CD4+ T-cells subsets that can further promote HIV replication and persistence (173). We also observed that pulmonary mucosal DN T cells of PLWH on ART expressed higher levels of HLA-DR and several cellular markers associated with HIV persistence (CCR6, CXCR3, and PD-1) compared to the blood (58). Importantly, CD3+CD4-CD8- DN T-cells from the BAL fluid of these participants harbored HIV DNA. Using the humanized bone marrow-liver-thymus mouse model, our group also observed higher infection frequencies of lung DN T-cells than those of the blood and spleen in both early and late HIV infection stages, meaning that apart from AMs and CD4 T-cells, HIV is also seeded in pulmonary mucosal DN T-cells early following infection and persists in these potential cellular HIV reservoirs even during long-term ART (58).

AMs pose yet a bigger challenge on our way to HIV reservoir eradication due to their abundance, longevity, and resistance to apoptosis (112, 174). As alluded to previously, HIV viral proteins remain detectable in BAL fluid of treated HIV-1 infected patients, as Collini and his group have demonstrated, which further underscores the role that AMs serve as HIV cellular reservoirs in the lung despite ART (112). Clayton and colleagues also show that HIV-infected macrophages are resistant to CD8 T-cell mediated killing, even more so than HIV-infected CD4 T-cells, which is associated with increased pro-inflammatory cytokine production (175). The relative importance of the lungs as viral reservoirs has also been highlighted by Horiike et al. in an SIV-infected Rhesus macaque model, where they found that the lungs and intestines of ART-treated animals had the largest burdens of SIV RNA, second to the lymphatic tissues (176). HIV persistence in the lung during ART has been further confirmed by Santangelo’s group using antibody-targeted positron emission tomography – a real-time, *in vivo* viral imaging method, showing that although lung viral signals are reduced after ART initiation they still remain detectable (177).

### Lung CD8 T-Cells Show Poor HIV-Specific Response

CD8 T-cells play a key role in clearance of virus-infected cells. Once differentiated into cytotoxic T-lymphocytes, they acquire several immunological effector functions. Their arsenal includes cytokines (IFN-γ, TNF-α, IL-2), cytotoxic granules containing perforin and granzymes, and the Fas ligand (178). These allow CD8 T-cells to kill infected target cells, activate and recruit phagocytes, and mediate pro-inflammatory processes, thus orchestrating and modulating the immune response (179, 180). As mentioned in the previous sections, chronic inflammatory environment and antigen stimulation leads impaired CD8 T-cell function in lungs of PLWH, rendering them susceptible to opportunistic infections. Although some of the effector functions of CD8 T-cells in the peripheral blood do recover after ART initiation, this is not the case for the CD8 T-cells in the lungs (181, 182). Moreover, elevation in CD8/CD4 ratio contributes to non-AIDS-related morbidity (183). These cells might also induce excessive expansion of other CD8 T-cells in the vicinity *via* T-cell receptor independent mechanisms, known as “bystander activation” (184, 185). Excessive expansion and immune activation consequently lead to accumulation of these functionally impaired CD8 T-cells displaying reduced proliferation, poor effector functions, and high expression of inhibitory receptors, such as PD-1, in the lung (184, 186, 187).

Impaired CD8 T-cell function may further contribute to lung reservoir persistence. Several studies have shown that these cells are required for HIV infection control. Once HIV-specific CD8 T-cells rise during acute infection, peak viremia begins to subside, meaning that these cells play a crucial part in viral control during primary infection (188, 189). Furthermore, SIV-infected Rhesus Macaques whose CD8 T-cells have been depleted, show increased plasma viremia, which is reversible with CD8 T-cell repopulation (190). Moreover, HIV mutants that can escape the CD8 T-cell response appear early during infection and persist, further demonstrating that there is a strong evolutionary pressure posed on the virus yielding CD8 T-cell escape highly advantageous (191, 192). In a recent study, our team has demonstrated that pulmonary CD8 T-cells show lower perforin expression *ex vivo* compared with blood CD8 T-cells, regardless of HIV or smoking status (193). Pulmonary CD8 T cells also showed significantly lower *in vitro* degranulation ability and less effective HIV-specific CD4 killing capacity than blood CD8 T cells, potentially contributing to a suboptimal anti-HIV immune response within the lungs.

## Interplay Between Chronic Inflammation and Viral Persistence Within the Lungs

### Persistent Lung Immune Dysfunction and HIV Persistence

Residual inflammation in PLWH is one of the biggest contributors to immune dysfunction, chronic co-morbidities, and replenishment of the viral reservoir even on ART. Several mechanisms have been put forth to explain HIV reservoir persistence after many years of ART treatment: poor ART penetration into deep tissues, residual replication, persistent cell stimulation due to residual antigen load, and CD8 T-cell exhaustion (194–196). Residual replication is of particular importance as it can create a self-perpetuating cycle where some of the virus will continue replicating, increasing viral antigen load locally (197, 198). In turn, this antigen load will stimulate nearby immune cells, activating their transcriptional machinery, which will produce more virus if that cell harbors intact and inducible viral DNA.

Studies have shown that the size of the viral reservoir is associated with residual levels of immune activation in ART-treated PLWH. Levels of both cell-associated RNA and proviral DNA have been positively correlated with frequencies of activated (CD38+HLA-DR+) and exhausted (PD-1+) CD4+ and CD8+ T cells (199, 200). Although most of these studies are typically done in the blood, the lungs of PLWH show a similar phenomenon. All lung T-cells show high frequencies of activation (HLA-DR) (17, 58, 193). Lung CD4 T-cells from treated PLWH also show higher frequencies of senescent cells (CD57), while CD8+ and double negative T-cells show high expression of PD-1 (17, 58, 193). These high levels of immune activation likely contribute to lung HIV reservoir persistence under ART, which our team has demonstrated to be larger in BAL CD4 T-cells compared to the blood (17). They also underline the presence of HIV-infected AMs in BAL fluid from PLWH on ART, which could induce chronic activation of the innate immune response in that tissue and contribute to T-cell dysfunction, as has already been shown in monocyte-derived macrophages (97, 201). Furthermore, Collini and colleagues were able to detect residual levels of HIV viral protein gp120 in BAL fluid of HIV-1-seropositive donors with median ART treatment time of 75 months (112). This finding is of particular importance, because the ability to detect HIV viral proteins in PLWH who have been on ART for over 6 years suggests that there is ongoing residual viral replication in that tissue that contributes to chronic pulmonary inflammation. In turn, chronic inflammation will consequently fuel HIV reservoir maintenance either through active viral replication or through proliferating latently infected cells, both of which are promoted by inflammatory environment, creating a vicious cycle that ensures HIV reservoir renewal (202–204).

### Dysbiosis of the Gut-Lung Axis Fuel Inflammation

Impaired lung immune function and HIV reservoir expansion are likely amplified through exacerbated inflammation caused by microbial dysbiosis in the lung and gut of PLWH (145, 205–208). Although the gastrointestinal and respiratory tracts are separate organs, they share a common mucosal immune system known as the gut–lung axis (209). Disruption of the gut microbiota has been linked with pulmonary diseases such as asthma and COPD (210, 211). Similarly, a well-balanced gut microbiome can help the lung immune system fight viral respiratory infections (212). Importantly, the lung and gut microbiomes do not exist in isolation from each other and prolonged immune activation, as seen in PLWH, affects both of them.

In PLWH, chronic inflammation isn’t just fueled by residual infection but also by damaged mucosal barriers and bacterial translocation, especially in the gut, which can further promote immune activation and HIV reservoir expansion in the lung. It is well known that HIV seeds into the gut within the first few weeks of infection and aggressively depletes memory CD4 T-cells in that tissue (213, 214). This is then followed by tight junction disruption between intestinal epithelial cells, leading to increased permeability, also known as a ‘leaky gut’, which might not be fully reversed with ART (215–217). This in turn leads to gastrointestinal dysbiosis and translocation of bacterial products, such as lipopolysaccharides, into the circulation leading to systemic innate immune activation (218–220). Notably, systemic markers of inflammation and innate defense have been associated with decreased lung function and pulmonary abnormalities in PLWH (221). Alterations in the pulmonary microbiome prior to ART initiation include decreased richness (α diversity) and increased change in species (β diversity) of microbial communities, as well as increased abundance of *Streptococcus* and *Tropheryma whipplei* in HIV-infected subjects compared to uninfected controls (144, 222). Although dysbiosis improve after treatment initiation, the lung microbiome is not always fully normalized even after effective ART. Levels of *Veillonella*, for instance, remain elevated in the lungs of PLWH even after 3 years of ART. Notably, *Veillonella* outgrowth has been previously associated with pulmonary inflammation in COPD patients characterized by higher lymphocyte and neutrophil count in BAL fluid and increased levels of exhaled nitric oxide (145, 223).

### Role of Smoking and Respiratory Co-Infections in HIV Persistence Within the Lungs

Smoking and co-infections further exacerbate pulmonary inflammation and thus could promote lung HIV reservoir persistence. Several research groups have shown that common co-infections in PLWH, such as hepatitis B, hepatitis C, and cytomegalovirus, are associated with higher circulating LPS levels, increased CD8 T-cell activation, and accelerated immunologic aging (224–226). *Mtb* infections, are especially detrimental to HIV-infected individuals. According to UNAIDS, PLWH are 19 times more likely to fall ill with tuberculosis (TB). In 2018, around 1.5 M people died from the disease, 251 000 of which died from TB that was AIDS-related, making it the leading cause of death in PLWH (227, 228). Notably, the risk of TB stays 4-fold higher in treated HIV+ individuals, despite ART and both diseases are characterized by chronic inflammation caused by failure to clear either pathogen (229). Importantly, *in vitro* studies show that macrophage infection with HIV-1 leads to impairment in IL-10 secretion in response to subsequent *Mtb* challenge, which is further confirmed by low IL-10 levels and high IL-1β levels in PLWH with tuberculosis, meaning that HIV can exacerbate pulmonary inflammation during TB infection even during ART. Furthermore, PLWH co-infected with *Mtb* have increased lung HIV viral load, and increased systemic HIV heterogeneity likely because more CD4 T-cells are recruited to pulmonary granulomas, which leads to accumulation of highly permissive HIV target cells, thus facilitating viral replication and cell to cell spread (230–233).

Smoking, which is highly prevalent in HIV-infected population, has also been shown to promote inflammatory immune environment (234). Neff and colleagues have recently documented increased expression levels CCR2, TLR4, CXCR4, and program death ligand 1 (PD-L1) by AMs in smokers regardless of HIV infection status (100). Importantly, higher TLR4 and CXCR4 levels were statistically significant only in HIV-infected smokers but not in seronegative nonsmokers and HIV+ nonsmokers, suggesting an additive inflammatory effect of HIV and smoking on AMs. They also highlight that the effect of CXCR4 upregulation on AMs in smokers should not be overlooked, since its upregulation in other contexts can lead to increased viral entry of X4-tropic HIV virus *in vitro* and increased viral evolution *in vivo* (100, 235).

### HIV and SARS-CoV-2 Infection

In the face of a pandemic, many contradictory statements have been made over the past two years regarding the relationship between HIV and SARS-CoV-2 infection. While some groups suggest that PLWH are more susceptible to SARS-CoV-2 infection and poor disease outcomes, other groups suggest that their immune suppression may prevent cytokine storm onset (236–241). Some groups even report that they do not see a significant difference in hospitalization rates or adverse outcomes caused by SARS-CoV-2 between PLWH and the general population (242, 243). In a meta-analysis, Ssentongo et al. examined studies published between January and December of 2020 and reported that the proportion of PLWH among SARS-CoV-2 infected patients in the cities was double compared to that found in the general population (244). Furthermore, they found that the risk of death in PLWH with SARS-CoV-2 infection was 80% greater than in HIV negative patients (244). However, the relationship between HIV and SARS-CoV-2 infection is challenging to decorticate due to many confounding factors, including multiple vulnerabilities and more often belonging to groups disproportionately affected by the pandemic than people without HIV infection (245).

Greater risk of SARS-CoV-2 infection and complications could be explained, in part, by accelerated immune aging and higher rates of co-morbidities in the HIV-infected population. Many of these co-morbidities, such as cardiovascular disease, diabetes, and cancer, are considered to be risk factors for severe SARS-CoV-2 infection outcomes independently of HIV infection (246, 247). Furthermore, given the immune perturbations of both innate and adaptive immune cells of the pulmonary mucosa, higher levels of pro-inflammatory cytokines, along with increased susceptibility of PLWH to respiratory infections, it is not surprising that they are also more likely to be infected with SARS-CoV-2 and suffer from a severe form of infection. Lastly, ART-discontinuation was also reported to be a risk factor for SARS-CoV-2 infection among PLWH, further stressing the importance of treatment-adherence in these patients (248). Importantly, preliminary data suggest that some PLWH, and especially those with low CD4 T-cell count below 250 cells/mm^3^ or detectable viral load, may exhibit a weaker humoral immune response to SARS-CoV-2 vaccination than persons without HIV infection, although results are mixed (249–251). Poor vaccine response in the setting of poorly controlled HIV or suboptimal CD4 T-cell recovery is well-recognized for many other pathogens, including pneumococcal pneumonia and influenza (252–255). The effects of SARS-CoV-2 on the lung HIV reservoir remain largely unknown. It is plausible that SARS-CoV-2-induced immune activation, similarly to what has been reported in PLWH with tuberculosis and other pulmonary infections, could stimulate viral replication and clonal expansion of HIV-infected cells but no studies have yet been published to support or refute this hypothesis (229, 256).

## Conclusion

In summary, ART does not fully restore lung immunity in PLWH, who continue to suffer from high burdens of pulmonary illnesses. Many different factors contribute to pulmonary immune perturbations in PLWH even during ART. Inflammation is the biggest driver of pulmonary pathologies and lung HIV reservoir persistence in these individuals. High blood flow, large pool of target cells, close cell-to-cell proximity, and small arteriole size likely contribute to lung HIV infection and spread. Furthermore, because the lung mucosa is continuously exposed to a large number of airborne antigens, these could further stimulate residual HIV replication and proliferation of infected cells. A true functional HIV cure will likely need to target many different factors to subdue both systemic and pulmonary inflammation such as promoting a healthy lung and gut microbiota, restoring epithelial integrity of the mucosal barriers, reducing microbial translocation, and controlling other co-infections.

## Author Contributions

YA wrote the first draft and constructed the figure. CC participated in the discussion and critically read and edited the manuscript. M-AJ designed the review and critically revised the manuscript. All authors contributed to the article and approved the submitted version.

## Funding

This work was funded by the Canadian Institutes of Health Research (CIHR) (grant 153082) and by CIHR-funded Canadian HIV Cure Enterprise (CanCURE) Team Grant HB2—164064, and in part by the *Réseau SIDA et maladies infectieuses du Fonds de recherche du Québec-Santé* (FRQ-S) to CC and M-AJ. YA is recipient of MSc scholarship from the FRQ-S. CC holds an FRQ-S Junior 2 Clinician-researcher salary award. M-AJ holds the CIHR Canada Research Chair tier 2 in Immuno-Virology.

## Conflict of Interest

The authors declare that the research was conducted in the absence of any commercial or financial relationships that could be construed as a potential conflict of interest.

## Publisher’s Note

All claims expressed in this article are solely those of the authors and do not necessarily represent those of their affiliated organizations, or those of the publisher, the editors and the reviewers. Any product that may be evaluated in this article, or claim that may be made by its manufacturer, is not guaranteed or endorsed by the publisher.
